# Association between alcohol and crack: Prevalence, effects, associated factors and experiences of combined use

**DOI:** 10.1371/journal.pone.0256414

**Published:** 2021-09-02

**Authors:** Naíde Teodósio Valois-Santos, Renata Barreto Fernandes de Almeida, Iracema de Jesus Almeida Alves Jacques, Daianny de Paula Santos, Keila Silene de Brito e Silva, Solange Aparecida Nappo, Ana Maria de Brito

**Affiliations:** 1 Departamento de Saúde Coletiva, Instituto Aggeu Magalhães, Fundação Oswaldo Cruz, Recife, Pernambuco, Brazil; 2 Curso de Psicologia, Centro Universitário UniFBV, Recife, Pernambuco, Brazil; 3 Programa de Pós-Graduação em Saúde Pública, Instituto Aggeu Magalhães, Fundação Oswaldo Cruz, Recife, Pernambuco, Brazil; 4 Núcleo de Saúde Coletiva, Centro Acadêmico de Vitória, Universidade Federal de Pernambuco, Vitória, Pernambuco, Brazil; 5 Centro Brasileiro de Informações sobre Drogas Psicotrópicas (CEBRID), Departamento de Medicina Preventiva and Departamento de Ciências Farmacêuticas, Universidade Federal de São Paulo, São Paulo, Brazil; Tufts, UNITED STATES

## Abstract

**Objective:**

To estimate the prevalence and factors associated with the effect of alcohol on crack cocaine use and to analyze experiences related to combined use. Materials and methods: sequential mixed methods (qualitative and quantitative) research, carried out between August 2014 and August 2015 with people who use crack. In the quantitative approach, a cross-sectional study was conducted with 1,062 participants. Factors associated with “alcohol use with the effect of increasing the effect of crack/crack craving” were estimated by multiple regression. In the qualitative approach, 39 interviews were conducted using Bardin’s content analysis technique.

**Results:**

871 (82.0%) participants reported consuming alcohol, among them, 668 (76.7%) used alcohol combined with crack: 219 (32.8%) reported feeling an effect of reduction in paranoia and/or crack craving and 384 (57.5%) reported feeling an increase in the effect of crack and in the craving to consume the drug. This relationship was also observed in the narratives of the people who use crack, with the possibility of a cyclic effect of consumption of the two substances. Those who related alcohol use to the effect of increasing crack craving (384) were more likely to use alcohol before crack (OR: 1.81; 95%CI: 1.13–2.89); to consume more than 20 stones daily (OR: 1.48; 95%CI: 1.01–2.16); to remain in abstinence from crack for less than one month (OR: 3.20; 95%CI: 1.91–5.35); to use dependence treatment services (OR: 1.85; 95%CI: 1.26–2.71); and to commit physical violence (OR:1.67; 95%CI:1.08–2.56).

**Conclusion:**

The findings of this study indicate that the modulation of the effect of alcohol use on crack cocaine depends on the moment when the drugs are consumed, and the use of alcohol before crack consumption is associated with characteristics that suggest a greater vulnerability to patterns of harmful crack use. Even though combined use is referred to as a way of reducing the negative effects of crack, the damage of this association may be greater than its possible benefits.

## Introduction

Alcohol is a psychoactive substance that is not usually perceived as a drug due to its sociocultural acceptability and outstanding position in the global market. However, excessive alcohol consumption has become one of the main problems of contemporary societies, mainly due to negative consequences to health and quality of life. Therefore, it is considered an important public health problem [1].

The Global Status Report on Alcohol and Health estimates that 237 million men and 46 million women suffer from disorders related to alcohol abuse. According to the report, in 2016, harmful use of alcohol resulted in 5.3% of all deaths and represented 5.1% of the global burden of diseases and injuries. It is a causal factor for more than 200 health problems, including injuries caused by accidents and violence, suicide, mental and behavioral disorders, noncommunicable diseases and infectious diseases, like tuberculosis and HIV/AIDS [2].

In Brazil, it is estimated that 66.4% of the population has consumed alcohol at some point in life and 30.1% consumed it in the last 30 days. This proportion is higher among men (38.8%) compared to women (21.9%) [3]. Among people who use crack cocaine, a prevalence of approximately 70% of alcohol consumption in the last 30 days has been found [4–7].

Combined use of alcohol and crack is a frequent practice in drug abuse scenarios and different functions and effects have been reported regarding this association: as a strategy to reduce paranoia, anxiety and fear, and also as something that “triggers” the outset of use of these substances, that is, it increases the desire to consume them [8–10]. Combined use can also be a strategy to potentiate the effect of crack, as the pharmacodynamic interaction between alcohol and cocaine significantly increases the systemic bioavailability of cocaine, producing higher chemical responses such as intense and long-lasting euphoria [8, 9, 11–13].

Combined use of alcohol and cocaine is also associated with greater vulnerability to HIV infection [14], greater facility to develop multiple and cross dependence, difficulty in adhering to and maintaining therapy [15, 16], difficulty in social reintegration, and with a higher number of episodes of criminal justice involvement, violence and imprisonment [17, 18]. Studies that enhance our understanding of crack and alcohol co-use can inform the design of tailored intervention approaches that reflect the lived experiences of people who use.

The ethnic, cultural, economic and social differences that divide Brazil in five distinct regions led the authors to develop the study in the Northeast, the region that presents the highest percentage of the population that uses crack regularly [6]. Pernambuco, the Northeastern state where the study was carried out, concentrates municipalities with significant numbers of people who use crack, and presents peculiarities in the culture of crack use, like consumption places and their dynamics [19], as well as forms to prepare the drug for consumption [20, 21]. For a comprehensive understanding, we used the mixed methods approach [22], which was employed with the purpose of obtaining data that complement each other and enable consistent and in-depth results on the subject investigated. In this perspective, the aim of this study was to estimate the prevalence and factors associated with the effect of alcohol use on crack use, and to analyze experiences related to the combined use of these substances in the Northeast region of Brazil, in four municipalities of the state of Pernambuco, including the state capital, Recife.

## Materials and methods

This study is part of the research “Vulnerability of crack cocaine users to HIV and other communicable diseases: a socio-behavioral and prevalence study in the state of Pernambuco”, funded by the Health Surveillance Department of the Ministry of Health (Notice 20/2013) [7] and approved by the research ethics committees of Fundação Oswaldo Cruz Pernambuco (CAAE-25250413.6.0000.5190) and Universidade Federal de São Paulo [23] (CAAE-33243514.3.0000.5505).

We employed the sequential mixed methods approach (qualitative and quantitative). Initially, quantitative data were collected and analyzed; subsequently, the stage of the qualitative approach was developed [22]. The final interpretation was performed in an integrated way, through convergence of numerical data with the detail and depth of qualitative data, which enabled us to be closer to the studied reality and to better understand the investigated problem [22, 24].

In the quantitative approach, a cross-sectional epidemiological study was carried out in a representative sample of people who use crack assisted by a program called *Atitude* (Attitude)—Comprehensive Care Program for Drug Users and their Families -, a public social protection service that aims to respond to the vulnerability situation associated with drug use. The Program is recognized for the excellence of the care it provides for people who use crack and other drugs [25, 26], prioritizing individuals who are in a situation of exposure to violence, face risk of death due to drug trafficking, and whose family bonds are weakened [27].

The sample was calculated based on the number of men (n = 1,594) and women (n = 355) assisted by the *Atitude* Program in 2013 and on HIV prevalence, totaling 766 men and 238 women [7]. The inclusion criteria were: being 18 years of age or older, using crack cocaine regularly (at least 25 days in the six previous months) [28], and not being in acute intoxication due to drug use.

Data collection was conducted between August 2014 and August 2015 in the four centers of the *Atitude* Program located in the cities of Recife, Jaboatão dos Guararapes, Cabo de Santo Agostinho and Caruaru. The sample was distributed among the centers proportionally to the number of men and women assisted in each of them in the year before data collection. A pilot study was carried out to test the procedures of the quantitative and qualitative stages.

People who use crack were invited to participate in the study during visits to the facilities of the *Atitude* Program centers. A consent document known as TCLE (*Termo de Consentimento Livre e Esclarecido*) was read to them and the objectives, procedures, risks and benefits were duly clarified. After the subjects signed the TCLE, a socio-behavioral questionnaire (S1 Appendix) was administered to them, containing questions about sociodemographic characteristics, drug use, access to health services, HIV infection, sex life, violence, and criminal justice involvement.

For data analysis, initially the frequency distribution of the main characteristics of the study population was calculated, according to the type of effect of alcohol use on crack use (increase in the effect of crack/crack craving; reduction in crack paranoia/craving), utilizing Pearson’s chi-squared test. Subsequently, “alcohol use with the effect of increasing the effect of crack/crack craving” was considered the outcome variable and its crude and adjusted associations with the questionnaire variables were analyzed by means of simple and multiple regression, respectively, using the backward selection procedure. The statistically significant variables in the bivariate analysis (p ≤ 0.20) were included in the initial, non-adjusted model, and the variables with p<0.05 were maintained in the final model. To quantify the strength of the association between the outcome variable and the independent variables, Odds Ratio (OR) values were calculated, as well as their respective confidence intervals (CI). The analyses were performed with the help of the software “Statistical Package for the Social Sciences”–SPSS, version 20.

In the second stage, the qualitative approach was employed to enhance our understanding of the social dynamics [29] related to the culture of crack use. The participants of the qualitative sample were recruited among those who had participated in the quantitative stage. Therefore, they met the inclusion criteria defined for that stage and another criterion necessary for the qualitative research: having a comprehensible speech. The technical team of the *Atitude* Program played the role of gatekeeper–facilitator of access to the study participants [30]–and selected, among those who used crack and participated in the quantitative stage, the ones who had a comprehensible speech.

The research objectives were explained to the selected participants and those who agreed to participate were referred to the researchers. After the TCLE was read to them, they were submitted to a semi-structured interview, whose questions were based on a script with topics previously prepared by the researchers (S2 Appendix). The script was based on the literature about the culture of crack use and addressed different themes.

Sample size was defined by theoretical saturation. This is a point defined as the interruption in the inclusion of new participants in the sample because the obtained data start presenting, according to the researcher’s appraisal, a certain redundancy or repetition; thus, the researcher believes it is not relevant to continue with data collection [31]. The point of theoretical saturation was achieved with 39 participants.

One of the researchers conducted all the interviews and one single professional was responsible for all the transcriptions, to avoid variations in the form of conduction and register, thus reducing possible biases in the analysis process. The interviewer has extensive experience of qualitative interviews and, additionally, she knew the population that is the object of this study, which contributed to establish the bond of trust between interviewee and interviewer, fundamental to the quality of a qualitative interview. Concerning data validation, the transcribed material was fully compared with the original audio version. To guarantee the participants’ anonymity, they were identified by names of precious stones in the presentation of their narratives.

In the analysis of the interviews, we used the content analysis technique, which was carried out through the analysis by thematic categories, based on the definitions proposed by Bardin [32]. The three recommended stages were performed: pre-analysis, exploration of the material and treatment of the results. The interviews were divided and the answers of all the interviewees to the same question were grouped. The thematic axes were identified and, at this point, with the aid of the software NVivo 10, the units of meanings that were important for each thematic axis were coded. These units were grouped by similarity, originating categories. This process was repeated more than once to refine the categories. The process of coding and formation of categories was developed by two other researchers (triangulation of researchers), ensuring data reliability and validity. The triangulation of methods (mixed methods) also contributed to data reliability and validity.

For the present study, “association of crack with other substances” was identified as the thematic axis, and the contents referring to the following categories of analysis are presented: “alcohol use to reduce paranoia and/or crack craving”; “alcohol use to “trigger” crack consumption”; “vicious circle of alcohol and crack consumption”.

As the phenomena most highlighted by the participants were paranoia and craving, it is important to explain their meaning. These phenomena are among the most evident symptoms related to crack cocaine consumption. Craving is characterized by an intense desire to consume the drug. It is considered a critical factor for the development of compulsive use and drug dependence, and for relapses after a period of abstinence [33]. Paranoia is the most severe psychiatric symptom among those produced by crack and is identified by paranoid states and persecutory delusions accompanied by olfactory, visual and auditory hallucinations. Both effects tend to worsen over time due to the sensitivity that the person who uses crack develops to these symptoms [34].

## Results and discussion

In the quantitative stage, 1,062 people were interviewed, a surplus of 58 participants in relation to the sample that had been initially calculated, in order to accommodate the demand of the people assisted by the *Atitude* Program. Of the 1,062 interviewees (819 men and 243 women), 871 (82.0%) reported consuming alcoholic drinks, and this proportion is higher among men (84.3%) compared to women (74.1%) (p = 0.02). Among those who consume alcohol, 668 (76.7%) reported using it associated with crack. Of them, the 219 individuals (32.8%) who stated feeling an effect of reduction in paranoia and/or in crack craving, and also the 384 individuals (57.5%) who reported feeling an effect of increase in the craving to consume the drug composed the sample for analysis, totaling 603 participants. A total of 40 (6.0%) participants who mentioned that alcohol has no effect on crack use were not included, nor were 25 (3.7%) participants who stated that alcohol use had the two effects described above or mentioned others, like avoiding the “dry mouth” effect or using crack to “make the *cachaça* (distilled alcoholic beverage *made* from fermented sugarcane juice, very popular in Brazil) come down”, that is, to reduce the effects of drunkenness.

The sociodemographic profile of the participants who reported that alcohol use increases the effect of crack craving (n = 384) was similar to the profile of those who reported that alcohol reduces paranoia and crack craving (n = 219). In both groups, the major part were men (78.8%) aged 25 years or older (71.0%; mean 29.3, median 27.0, SD 8.0), with brown skin color (65.0%), single/separated/widowed (82.1%), with less than eight years of schooling (71.0%), living on the streets, in shelters run by the government, hospitals, dependence treatment services or in prison on the 30 previous days (62.5%), with income lower than one minimum salary (52.4%; mean R$ 1,508.9; median R$ 700.0; SD R$ 6,403.2) (Table 1). This profile is similar to the one found in a national survey on crack cocaine use [6]; however, the frequency of individuals with no fixed address and who live alone is higher in our study, which highlights the high social vulnerability of the people who use crack assisted by the *Atitude* Program.

**Table 1:** Socio-demographic characteristics of people who use crack that reported some effect of alcohol on crack craving, assisted in the *Atitude* Program, Pernambuco, August 2014 to August 2015.

Alcohol effect on crack craving								
Variables	Reduction (n = 219)		Increase (n = 384)		χ2	P-value	Total (N = 603)	
	n	%	n	%			n	%
**Sex**					3.107	0.07		
Male	164	74.9	311	81.0			475	78.8
Female	55	25.1	73	19.0			128	21.2
**Age group (years)**								
18 to 24	60	27.4	115	29.9	0.440	0.50	175	29.0
25 or older	159	72.6	269	70.1			428	71.0
**Skin color**					1.679	0.64		
White	38	17.4	61	15.9			99	16.4
Black	31	14.2	66	17.2			97	16.1
Brown	143	65.3	249	64.8			392	65.0
Others (yellow and indigenous)	07	3.2	08	2.1			15	2.5
**Marital status**					0.154	0.69		
Single, separated or widowed	178	81.3	317	82.6			495	82.1
Married or lives with partner	41	18.7	67	17.4			108	17.9
**Considers him/herself religious**					2.476	0.11		
Yes	176	80.4	287	74.7			463	76.8
No	43	19.6	97	25.3			140	23.2
**Level of schooling (in years)**					1.177	0.27		
< 8 (never went to school to incomplete junior high school)	150	68.5	279	72.7			429	71.1
≥ 8 (complete junior high school to complete higher education)	69	31.5	105	27.3			174	28.9
**Place where lived/slept in the 30 previous days**					0.136	0.71		
No fixed address and others^a^	137	63.4	234	61.9			371	62.5
Lives in his/her own house, in partner’s, friends’ or in a rented house	79	36.6	144	38.1			223	37.5
**Current work situation**					3.578	0.06		
Does not work	84	38.9	175	46.9			259	44.0
Works (formally or informally)	132	61.1	198	53.1			330	56.0
**Individual monthly income**					31.01	0.08		
< 1 minimum salary^b^	125	57.1	191	49.7			316	52.4
≥ 1 minimum salary	94	42.9	193	50.3			287	47.6

^a^ Living on the streets, in shelters run by the government, hospitals, treatment services or in prison on the 30 previous days.

^b^ In 2014, the minimum salary corresponded to R$ 724.00.

Early onset of alcohol use (before 13 years of age) was reported by 174 (28.9%) participants (Table 2) and, among them, 31 (17.9%) also reported early onset of crack use (non-tabulated data). The mean age at onset of alcohol use was 14.0 years (median 14.0, SD 3.0), while the mean age at onset of crack use was 19.8 years (median 18.0, SD 7.3) (non-tabulated data). On the 30 previous days, 317 (59.4%) people who use crack reported having used crack on a daily basis, while 191 (36.5%) reported having used alcohol every day in the same period (Table 2). Among those who reported using crack daily, 66.8% also used alcohol everyday (non-tabulated data). The average number of stones consumed per day was 10.5 (median 2.0, SD 26.5) (non-tabulated data).

**Table 2:** Characteristics of crack consumption among participants assisted in the *Atitude* Program, Pernambuco, August 2014 to August 2015.

Alcohol effect on crack craving								
Variables	Reduction (n = 219)		Increase (n = 384)		χ2	P-value	Total (N = 603)	
	n	%	n	%			n	%
**Age at onset of alcohol use (years)**					0.912	0.63		
5 to 12	62	28.3	112	29.2			174	28.9
13 to 15	92	42.0	171	44.6			263	43.7
> 15	65	29.7	100	26.1			165	27.4
**Moment in which uses alcohol**					59.37	**<0.01**		
Simultaneously with crack use	57	26.0	119	31.0			176	29.2
After using crack	110	50.2	81	21.1			191	31.7
Before using crack	52	23.7	184	47.9			236	39.1
**Used alcohol every day in the previous month**					0.025	0.87		
Yes	70	36.1	121	36.8			191	36.5
No	124	63.9	208	63.2			332	63.5
**Age at onset of crack use (years)**					2.26	0.32		
5 to 12	15	6.9	31	8.1			46	7.7
13 to 15	33	15.3	75	19.6			108	18.1
> 15	168	77.8	276	72.3			444	74.2
**Crack use in an intense way (all night)** ^a^					0.00	0.97		
Yes	206	94.1	361	94.0			567	94.0
No	13	5.9	23	6.0			36	6.0
**Forms of crack consumption** ^b^					0.919	0.33		
Compulsive	163	74.4	299	77.9			462	76.6
Controlled	56	25.6	85	22.1			141	23.4
**Used crack every day in the previous month**					0.007	0.93		
Yes	80	40.4	199	59.2			317	59.4
No	118	59.6	137	40.8			217	40.6
**Number of consumed stones** ^b^					5.22	**0.02**		
Up to 20 stones	134	61.2	198	51.6			332	55.1
≥ 21 stones	85	38.8	186	48.4			271	44.9
**Obtained crack participating in drug sale or distribution** ^c^					3.88	**0.04**		
Yes	27	12.3	71	18.5			98	16.3
No	192	87.7	313	81.5			505	83.7
**Largest period of abstinence from crack**					12.03	**<0.01**		
Less than one month	31	14.2	101	26.3			122	21.7
More than one month	188	85.8	283	73.7			440	78.3
**Underwent treatment for crack dependence at some point in life**					0.547	0.46		
Yes	94	42.9	153	39.8			247	41.0
No	125	57.1	231	60.2			356	59.0

^a^ Spent more than one day using crack intensely.

^b^ Data referring to the period before admission to the *Atitude* Program.

^c^ Means that do not involve money to buy crack.

The results of the quantitative stage show the association of crack with alcohol depends on the moment in which the latter is consumed. Those who use alcohol after crack reported, more frequently, an effect of reduction in crack craving, while the use of alcohol before crack was associated with an increase in the effect of crack/crack craving, with a statistically significant difference (p-value <0.001) between the moment of alcohol use and the effect caused (Table 2)

The results obtained in the qualitative stage reinforce and deepen the findings of the quantitative research, that is, the effects of the association between crack and alcohol were related to the moment in which the person who uses crack consumes alcohol. In the narratives produced through the qualitative interviews, the participants reported that using alcohol after crack had a “calmative” function, tranquilizing or reducing the negative effects experienced with the use of crack. On the other hand, when they consume alcohol before crack, they reported the “triggering” function, that is, alcohol triggers the desire to use crack.

The narratives below refer to alcohol, consumed after crack, as an agent that reduces the paranoia symptoms caused by crack.

Alcohol use to reduce paranoia and/or crack craving

*I started using alcohol*, *too*, *because of crack use*. *When crack ends in a moment I need to use something*, *I run to alcoholic drinks*, *I run to pills*, *I run to* loló (clandestine preparation made of chloroform and ether; different compositions can be found, used only for abuse purposes), *to other kinds of drug (*…*)*. *Alcohol reduces more the effect of crack*, *it prevents the effect of crack from exploding*, *then it doesn’t cause that paranoia in which I run back and forth*, *trying to see things* (Agate).

Cachaça *calms me down*, *makes me go back to my normal state*, *there’s no alteration*, *there is no bug*. *I smoke a joint*, *I have one drink and everything’s fine*, *you know*? (Amethyst).

*Alcohol stops that sensation of looking one way and the other*. *(*…*) that there are people looking*, *staring*, *there are people behind you (*…*)*. *When we’re smoking crack and the effect fades quickly*, *we drink alcohol and it turns us on immediately*. *(*…*) we feel happy*, *we smile*, *we play*, *we dance*, *we have fun*. *Crack doesn’t do this*. *It causes frustration*. *I started to smoke crack before I consumed alcohol*, *then I realized that when I start drinking*, *that frustration goes away* (Alexandrite).

Ribeiro, Sanchez and Nappo [35] also reported on the effect of alcohol as a substance capable of reducing the transitory paranoia effects and mitigating fear and anxiety. In addition, they found it is capable of serving as a “calmative” for the craving effect, reducing the need to use more crack.

In addition to the tranquilizing effect, in the reports below we see that alcohol helps to mask the appearance of the person under the effect of crack—sensation of fear, wide eyes and frightened appearance.

*Alcohol reduces more the effect of crack*, *it makes you happier and prevents hallucinations*, *of things*, *of bad things*. *You become happier and you don’t stay there*, *interned* [the term “interned” is used to refer to the situation where a person stays at a place for a very long time, using crack continuously]. *When you use only crack*, *you just want to stay there*. *I’m so ashamed of people*, *because I’m under the effect of crack and I think it’s a very frightful scene* (Gold).

*Crack makes us nervous*, *anxious*, *scared; alcohol doesn’t; it relaxes us*, *it makes us calm*, *it removes the intensity of crack*. *Now*, *if you’re using just crack*, *you get frightened*, *you walk looking one way and the other*. *I think that*, *when you use alcohol*, *you reduce that intensity you have in the use of pure crack*. *That’s why I use both* (Coral).

On the other hand, alcohol consumption before crack use appears to have a “triggering” effect, stimulating the use of crack. In the experience of some people who use crack, consuming alcohol means using crack right after it, as Ribeiro, Sanchez and Nappo [35] and Leite, Oliveira and Cruz [36] found in the studies they developed.

The narratives below reveal the desire of using crack after alcohol consumption.

Alcohol use to “trigger” crack consumption

*To sleep on the street*, *you must drink and I can’t drink*: *when I drink*, *I get crazy*, *I have serious problems with alcohol*. *If I drink*, *oh my God*, *while I don’t drink I don’t use drugs*, *and to sleep on the street*, *you must get drunk*, *sober people don’t sleep on the street*. *Because if you die*, *you sleep and don’t feel (*…*)*. *I only use it after I drink*, *when I’m sober I don’t feel like using it* (Aquamarine).

*If I drink*, *I have to smoke crack; if I’m drinking today*, *you can say to me*: *today you’ll smoke crack*. *Because alcohol calls for crack*, *in me it causes an immense desire to smoke* (Lapis lazuli).

What Aquamarine reported bolsters what was obtained in the quantitative stage, through which it was possible to observe a greater proportion of daily use of alcohol among individuals who had spent the 30 previous days living on the streets (74.1%) when compared to those who lived in rental housing or had their own home (25.9%) (p-value<0.001) (non-tabulated data). Desire for alcohol can emerge in response to constant stressful situations [37], as it is the case of homeless people. These findings reveal that daily use of alcohol is a characteristic related to social marginalization, aggravated by its “triggering” effect for crack cocaine consumption. Thus, life conditions, including the physical and social environment, play a fundamental role in the profile of drug use [38].

To some participants, alcohol use has both effects—it increases the desire for crack (increases craving) and prolongs the pleasant effect (reduces craving) of crack. When using alcohol, these interviewees feel the need to use crack and, subsequently, due to the negative and paranoid effects of crack, they use alcohol again to feel better. This vicious circle of alcohol and crack consumption can last several days, causing greater consumption of both substances, contributing to a neuroadaptation of the dopaminergic system [39], and, consequently, to alcohol tolerance and dependence [40].

Vicious circle of alcohol and crack consumption

*You take two*, *three drinks of* cachaça *and it reduces the adrenaline*, *the sensation of fear*. *It is the* cachaça *that reduces the effect*. *Alcohol comes after the crack*, *but sometimes*, *I drink* cachaça *before*, *then the desire hits me and I smoke crack*. *Then*, *when I smoke it*, *I drink* cachaça *and it reduces the effect* (Garnet).

*It tranquilizes me*, *I don’t get paranoid (*…*) you become softer (*…*) after smoking I need to drink (*…*) if I drink first*, *I smoke*, *or if I smoke first*, *I have to drink* (Aventurine).

In the present study, intense use, monthly frequency and the form in which participants evaluate crack consumption were not different between the individuals who reported that alcohol has the effect of reducing crack craving and those who reported that alcohol has the effect of increasing crack craving. However, we noticed that the group that reported alcohol use to increase the effect of crack craving, when compared to the group that reported using it to reduce paranoia and/or craving, presented a higher frequency of daily consumption of more than 20 stones; of involvement in drug trafficking to obtain crack cocaine; and of a period of abstinence from crack use lower than 30 days. All these differences are statistically significant (Table 2).

No statistically significant differences were found between the two above-mentioned groups concerning use of condoms in the last sexual relationship and self-perception of the likelihood of being infected with HIV. However, the people who reported that alcohol has the effect of increasing crack craving, when compared to those who reported that it reduces paranoia and/or craving, presented a higher frequency of access to dependence treatment services and of practice of drug-related physical violence with the use of guns or cold weapons at some point in life. These differences are statistically significant (Table 3).

**Table 3:** Characteristics of the utilization of health and treatment services, sexual behavior, self-perception of the likelihood of being infected with HIV, violence and criminality among people who use crack assisted in the *Atitude* Program, Pernambuco, August 2014 to August 2015.

Alcohol effect on crack craving								
Variables	Reduction (n = 219)		Increase (n = 384)		χ2	P-value	Total (N = 603)	
	n	%	n	%			n	%
**Used health services in the 12 previous months**					2,346	0,12		
Yes	144	64.7	224	58.3			365	60.6
No	77	35.3	160	41.7			237	39.4
**Used services to treat dependence on alcohol, crack or other drug in the 12 previous months**					9.123	**<0.01**		
Yes	83	38.2	196	51.0			279	46.4
No	134	61.8	188	49.0			322	53.6
**Use of condom in the last sexual relationship**					2.28	0.13		
Yes	94	44.1	144	37.8			238	40.1
No	119	55.9	237	62.2			356	59.9
**Self-perception of the likelihood of being infected with HIV**					5.022	0.08		
None	67	31.6	153	40.2			220	37.1
Low	104	49.1	154	40.4			258	43.5
High	41	19.3	74	19.4			115	19.4
**Result of the last HIV test (self-stated)**					0.000	1.00		
Positive	08	5.0	12	5.0			20	5.0
Negative	152	95.0	228	95.0			380	95.0
**Committed physical violence at some point in life and it was related to drug use** ^a^					5.04	**0.02**		
Yes	51	23.4	123	32.0			174	28.9
No	167	76.6	261	68.0			428	71.1
**Was arrested at some point in life**					2.134	0.14		
Yes	101	46.5	201	52.8			302	50.5
No	116	53.5	180	47.2			296	49.5

^a^ Any kind of physical violence committed with guns or cold weapons (revolver, knife, stiletto, glass shard or other sharp objects) was considered.

To assess the influence of different aspects of vulnerability on the occurrence of alcohol use with the effect of increasing crack craving, a multivariate logistic regression was performed. Initially, the factors that were associated (p-value ≤ 0.20) with alcohol use with the effect of increasing crack craving were included, except the variable “current work situation”, because it associates strongly with “individual monthly income”. Based on the variables that remained in the final model (p-value<0.05), we found that the individuals who reported that alcohol use increased crack craving were more likely to use alcohol before crack (OR: 1.81; 95%CI: 1.13–2.89); to consume more than 20 stones daily (OR: 1.48; 95%CI: 1.01–2.16); to remain in abstinence from crack for less than one month (OR: 3.20; 95%CI: 1.91–5.35); to use dependence treatment services (OR: 1.85; 95%CI: 1.26–2.71); and to commit physical violence (OR: 1.67; 95%CI: 1.08–2.56). On the other hand, those who consumed alcohol after crack were less likely to report the increase in crack craving (OR: 0.31; 95%CI: 0.19–0.50) (Table 4). The result of the Lemeshow test showed that the data adjusted adequately to the model (p = 0.396), which could explain correctly 70.2% of the cases.

**Table 4:** Odds Ratio and confidence interval for alcohol use with the effect of potentiating crack craving among people who use crack assisted in the *Atitude* Program, Pernambuco, August 2014 to August 2015, according to the multivariate regression model.

Variables	Univariate Regression		Multivariate	
	OR (95%CI)	P-value	OR (95%CI)	P-value
**Main exposure variable**				
**Number of consumed stones**				
Up to 20 stones	1.48 (1.05–2.07)	0.02	1.48(1.01–2.16)	0.04
≥ 21 stones	**-**		-	
**Co-variables**				
**Sex**				
Male	1.42 (0.96–2.12)	0.07		
Female	-			
**Considers him/herself religious**				
Yes	-			
No	1.38 (0.92–2.07)	0,11		
**Individual monthly income**				
< 1 minimum salary	-			
≥ 1 minimum salary	1.34 (0.96–1.87)	0.08		
**Obtained crack illegally**				
Yes	1.61 (1.00–2.60)	0.05		
No	-			
**Largest period of abstinence from crack**				
Less than one month	2.16 (1.39–3.37)	<0.001	3.20(1.91–5.35)	<0.001
More than one month	-		-	
**Moment in which uses alcohol**				<0.001
Simultaneously with crack use	-		-	
After using crack	0.35 (0.23–0.54)	<0.001	0.31(0.19–0.50)	<0.001
Before using crack	1.69(1.09–2.63)	0.01	1.81(1.13–2.89)	0.01
**Used health services in the 12 previous months**				
Yes	-			
No	1.30 (0.92–1.84)	0.12		
**Used services to treat dependence on alcohol, crack or other drug in the 12 previous months**				
Yes	1.68(1.19–2.36)	<0.001	1.85(1.26–2.71)	<0.001
No	-		-	
**Use of condom in the last sexual relationship**				
Yes	-			
No	1.30(0.92–1.82)	0.13		
**Self-perception of the likelihood of being infected with HIV**				
None	1.54(1.05–2.25)	0.02		
Low	-			
High	1.21(0.77–1.92)	0.39		
**Committed physical violence at some point in life**				
Yes	1.55(1.06–2.26)	0.02	1.67(1.08–2.56)	0.01
No	-		-	
**Was arrested at some point in life**				
Yes	1.28(0.91–1.79)	0.14		
No	-			

The quantitative results are aligned with the interviewees’ discourses, showing that the moment of alcohol use, with regards to the moment of crack use, appears to play a significant role in the subjective and behavioral effects of crack. Although the use of alcohol after crack was related to a reduction in paranoia and craving, this strategy, identified by people who use crack as a way of diminishing the negative effects of crack (as it has been observed also with the use of marijuana [14, 35, 41], may stimulate a desire for crack, therefore inciting crack craving. Thus, alcohol might induce a vicious consumption circle of the two substances, contributing to a greater risk of alcohol and crack dependence [42], overdose [43], and other health damages caused by the use of crack cocaine [44].

Based on the multivariate analysis, we can observe that alcohol use before crack is associated with consumption of a higher number of stones and with a shorter period of abstinence from crack; in addition, it is associated with greater vulnerability to commit physical violence and with looking for treatment services. Crack craving leads to a pattern of compulsive use of the drug, contributing to increase dependence [33]. Consequently, in face of the urgency to obtain it, people who use crack can get involved in risk situations, with aggressive and violent behaviors [34]. However, we found that, in the context of compulsive crack use associated with violence, the participants often reported looking for dependence treatment services.

This research has some limitations concerning its design, as it is a cross-sectional observational study, which, a priori, does not allow establishing a causal relationship. Even though we performed the investigation respecting the temporality of the facts, there is the problem of the participants’ memory bias.

Another limitation is the fact that the components of the studied population have a very similar socioeconomic profile, as they were recruited in a public social protection service. On the other hand, the characteristics identified in the majority of the participants (man aged 25 years or older, with brown skin color, single, with low level of schooling and low income) (Table 1) are similar to those presented in the profile of people who use crack in Brazil, described in the pioneering national study carried out by Fundação Oswaldo Cruz in partnership with Brazil’s National Department of Drug Policies [6], which enabled the extrapolation of our findings.

## Final remarks

This study was developed in a population of the Northeastern region of Brazil, which concentrates approximately 40% of the population that uses crack regularly in the country and has characteristics that are different from those of the other Brazilian regions in relation to social, cultural, ethnic, and economic parameters, and to the culture of crack use. These differences might influence our results regarding the reports on the influence of the alcohol effect on crack effect, depending on the moment of their consumption. However, the findings were analogous to those available in the literature, showing that, depending on the theme, some aspects of the culture of crack use seem to be similar in different places, independently of regional characteristics.

In short, the findings of this study indicate that the effect of alcohol use can interfere, in different ways, in the effects of crack, depending on the moment in which the drugs are consumed. When alcohol was consumed before crack, it was related to increased crack craving and a consumption of a greater amount number of stones. However, when alcohol was consumed after crack, it was often characterized as a way of reducing the paranoia and/or craving caused by crack.

We highlight, as one of the findings of this study, that use of alcohol before crack consumption is associated with characteristics that suggest a greater vulnerability to patterns of harmful crack use, such as staying in abstinence for a shorter time, consumption of higher number of stones per day and higher frequency of reports on committing aggressive acts. On the other hand, people who use alcohol before crack also reported a greater demand for dependence treatment services, which shows the need of public facilities to shelter and offer treatment to people with intense consumption.

Employment of the mixed method approach used currently provided evidence for linking particular patterns of alcohol consumption with the expected outcome of crack cocaine use. For instance, the perceived stigma of crack cocaine use might be attenuated by alcohol consumption. Alcohol use may also be implemented during or after crack cocaine use in order to prevent some of the negative effects associated with cocaine, such as anxiety or psychosis. Unfortunately, it appears that the negative outcomes and the perpetuation of drug taking may be amplified by the combination of alcohol and crack cocaine. We hypothesize that such interactions induce a vicious cycle for the consumption of the two substances, which is strengthened by the higher frequency of daily use of crack found among those who use alcohol on a daily basis. These findings reinforce the need to enhance comprehensive alcohol treatment and harm reduction approaches.

## Supporting information

S1 Appendix Questionnaire. (DOCX)

S2 Appendix Interview guide. (DOCX)
