# Supplementary material for: Association between alcohol and crack: Prevalence, effects, associated factors and experiences of combined use
Source: PLoS One. 2021 Sep 2;16(9):e0256414. doi: 10.1371/journal.pone.0256414 (PMC8412309; doi:10.1371/journal.pone.0256414)
Supplement: S1 Appendix — (DOCX) [file pone.0256414.s001.docx]

**Interviewer: Date of interview:** _____/_____/______

**Start time: End time:**

**City:** (1) Recife (2) Jaboatão (3) Cabo (4) Caruaru

**Interview venue (*ATITUDE*)**: (1) Support (2) Intensive (3) Other:

**Legal name:**

**Name-in-use:**

**Name of mother:**

**Enrolment in *ATITUDE*: Length of assistance in *ATITUDE*:** _________ months

**Questionnaire no.:**

*Notes:*

*1) The questionnaire administered in the research has 10 sections and 171 questions. For the development and data analysis of the paper “Association between alcohol and crack: Prevalence, effects, associated factors and experiences of combined use”, we selected the questions listed below, according to their presentation in the paper’s tables.*

*2) The acronyms NQR* (não quis responder) *and NSNL* (não sabe/não lembra) *used in the questionnaire mean ‘did not want to answer’ and ‘does not know/does not remember’, respectively.*

**(Table 1)**

**Q.56 Does alcoholic beverages have any function / effect in relation to your crack use?** *(Mark all the applicable answers)*

(1) Decrease craving and / or paranoia

(2) Increase crack effect / crack

(3) Other_______________

(4) NSNL

(5) No

**Q.03 What is your date of birth**? ________/________/_______ or **Age:** _____________ **years**

**Q.04 What is your sex** (biological sex/the sex with which you were born)**?**

1. Male
2. Female
3. Hermaphrodite

**Q.06 How do you classify yourself in relation to your skin color or race?**

1. White
2. Black
3. Yellow/Asian
4. Brown
5. Indigenous

**Q.07 What is your marital status?**

1. Single (never married / never lived with partner / never was in a stable union)
2. Married/ Stable union / Lives with partner
3. Separated/ Divorced
4. Widowed

**Q.09 Do you consider yourself religious?**

1. Yes
2. No **=> Go to Q.11**

(88) NQR **=> Go to Q.11**

(99) NSNL **=> Go to Q.11**

**Q.11 What is your level of schooling?**

1. Never went to school
2. Learned how to read and write
3. Incomplete elementary school
4. Complete elementary school
5. Incomplete junior high school
6. Complete junior high school
7. Incomplete high school
8. Complete high school
9. Incomplete technical school
10. Complete technical school
11. Incomplete higher education
12. Complete higher education

(88) NQR

(99) NSNL

**Q.16 On the last 30 days, where did you live or sleep/spend the majority of nights?**

(1) Own apartment/house or family’s apartment/house

(2) Rented apartment/house/room

(3) Friend’s/acquaintance’s house

(4) Occupied apartment/house (squatting)

(5) Private hotel/motel/boardinghouse/hostel room

(6) Public or philanthropic shelter/hostel

(7) Hospital (without including hospitalization for drug dependence treatment)

(8) Service that provides treatment/healthcare for people who use drugs => Answer Q.16.1

(9) Prison/police station

(10) Street

(11) Other ______________________________________________________

(99) NSNL

**Q.19 What is your main occupation/job now?** *(Or before you were sheltered in a health institution/social protection facility, spent the night in an institution, was hospitalized or in prison).* INTERVIEWER: in the case of informal or illegal work, please characterize duration and periodicity so that we can establish if the person works on their own/autonomously or if it is sporadic work/odd job.

1. Public servant
2. Registered employee (subject to the Brazilian Consolidation of Labor Laws)
3. Unregistered employee (not subject to the Brazilian Consolidation of Labor Laws)

(What job?_______________________________________ )

1. Works on his/her own/autonomous (What job? _____________________________________ )
2. Sporadic works/odd jobs (What job? ____________________________________________ )
3. Employer
4. Student
5. Housewife
6. Retired
7. Not working at the moment
8. Other ________________________________________________

(88) NQR

**Q.22 On the last 30 days, how much money did you earn or receive?** R$ ______________.00

**(Table 2)**

**Q26. How old were you when you started using: (years)**

a) Alcohol?

f) Crack?

**Q.55 Do you consume alcoholic beverages with crack?** *(Mark the main, most common, form)*

(1) I don’t drink **=> Go to** **Q.57**

(2) I drink, but independently of crack use (on different moments of the day) **=> Go to** **Q.57**

(3) Simultaneously with crack use

(4) I drink **after** I use crack

(5) I drink **before** I use crack

**Q28. On how many of the last 30 days did you use:**

a) Alcohol?

f) Crack?

**Q.32 Have you ever spent more than 1 day using crack in an intense way (during all day and night)?**

(1) Yes

(2) No

**Q.34 Before you had contact with the *ATITUDE* Program, which of the forms below was more similar to your way of using crack?**

*(Read all the options)*

(1) Used normally the same amount/controlled the amount, that is, decided the amount that would be used

(2) Used while he/she had the drug at hand/was able to obtain the drug/did not control the amount used (used compulsively)

**Q.36 How many crack stones (on average) did you normally consume before you had contact with the *ATITUDE* Program? ___________**

**Q.48 On the last 30 days, did you use any of the means below, which do not involve money, to obtain crack?**

*(If he/she is sheltered in a health institution/social protection facility, spends the night at an institution or is hospitalized, ask about the 30 previous days)*

*Mark the answers that are applicable to what happens normally.*

1. Fixing or cleaning things, helping someone with legal work
2. Participating in/helping with illegal work, such as piracy, unrelated to drug trafficking
3. Preparing for trade, selling, participating in some way in drug sale or distribution
4. Trading for sex
5. Trading for things (not drugs)
6. Trading for another drug
7. Other __________________________________________________
8. None of these methods; I only obtain crack when I trade it for money.

**Q.49 Since you started using crack, which was the largest period during which you did not use it?**

(1) Never stopped (never spent a period without using it, considering his/her frequency of use) **=>Go to Q.53**

(2) 1 to 2 weeks **=>Go to Q.53**

(3) 3 to 4 weeks **=>Go to Q.53**

(4) More than 1 month

(5) More than 1 year

**Q.66 Which drugs have you undergone treatment for?**

(1) Alcohol

(2) Tobacco

(3) Marijuana

(4) Cocaine

(5) Crack

(7) Inhalants

(6) Other(s)_____________________________________

**(Table 3)**

**Q.62 Which of the services below did you use in the last 12 months? *(Read all the options)***

*(If he/she is sheltered in a health institution/social protection facility, spends the night at an institution or is hospitalized, ask about the 12 previous months)*

| - - 1. Social Work Services *(except *ATITUDE*) | (1) Yes | (2) No | (99) NSNL |
| --- | --- | --- | --- |
| - - 1. Social Security (allowances, insurance and other benefits) | (1) Yes | (2) No | (99) NSNL |
| - - 1. Services that provide free meals | (1) Yes | (2) No | (99) NSNL |
| - - 1. Programs to find work, employment and income | (1) Yes | (2) No | (99) NSNL |
| - - 1. Healthcare Unit or Center, Outpatient Clinic | (1) Yes | (2) No | (99) NSNL |
| - - 1. Hospital (hospitalization due to reasons other than drug dependence) | (1) Yes | (2) No | (99) NSNL |
| - - 1. Emergency services | (1) Yes | (2) No | (99) NSNL |
| - - 1. Street social work | (1) Yes | (2) No | (99) NSNL |
| ***Dependence treatment services*** | | | |
| - - 1. Psychiatric Hospital | (1) Yes | (2) No | (99) NSNL |
| - - 1. General Hospital | (1) Yes | (2) No | (99) NSNL |
| - - 1. Psychosocial Care Center | (1) Yes | (2) No | (99) NSNL |
| - - 1. Shelter/Therapeutic Hostel | (1) Yes | (2) No | (99) NSNL |
| - - 1. Private Specialized Clinic | (1) Yes | (2) No | (99) NSNL |
| - - 1. Therapeutic Community | (1) Yes | (2) No | (99) NSNL |
| - - 1. University-based Service, Outpatient Clinic | (1) Yes | (2) No | (99) NSNL |
| - - 1. Mutual Help Group | (1) Yes | (2) No | (99) NSNL |
| - - 1. Other ***______________________________________________*** | (1) Yes | (2) No | (99) NSNL |

**Q.127 Did you use a condom in your last sexual relationship?**

1. Yes
2. No
3. NQR

(99) NSNL

**Q.93 IF THE PERSON DID NOT REPORT A POSITIVE HIV TEST RESULT:**

**Nowadays, how do you evaluate your chances of getting infected with HIV?**

**IF THE PERSON REPORTED A POSITIVE HIV TEST RESULT:**

**Nowadays, how do you evaluate your chances of getting infected with HIV again?**

*(Read all the options)*

1. None
2. Low chance
3. High chance
4. NQR
5. NSNL

**Q.91 What was the result of your last HIV test?**

1. Positive
2. Negative
3. Indeterminate
4. Got tested but did not see the result

(88) NQR

(99) NSNL

**Q.162 Have you ever wounded anyone with a knife, stiletto, glass shard, revolver or other object?**

*(Mark all the applicable answers)*

1. Yes, and it was related to drug use
2. Yes, but with no relation to drug use
3. No
4. NQR
5. NSNL

**Q.170 Have you ever been arrested?**

1. Yes
2. No
3. NQR
