# Supplementary material for: Association between alcohol and crack: Prevalence, effects, associated factors and experiences of combined use
Source: PLoS One. 2021 Sep 2;16(9):e0256414. doi: 10.1371/journal.pone.0256414 (PMC8412309; doi:10.1371/journal.pone.0256414)
Supplement: S2 Appendix — (DOCX) [file pone.0256414.s002.docx]

**Interviewer: Date of interview:** _____/_____/______

**Start time: End time:**

**City:** (1) Recife (2) Jaboatão (3) Cabo (4) Caruaru

**Interview site (*ATITUDE*)**: (1) Support (2) Intensive (3) Other:

**Legal name:**

**Name-in-use:**

**Name of mother:**

**Enrolment in *ATITUDE*: Length of assistance in *ATITUDE*:** _________ months

**Questionnaire no.:**

*Note: The semi-structured interview conducted in the research has 54 guiding questions distributed among 15 thematic sections. For the development and data analysis of the paper “Association between alcohol and crack: Prevalence, effects, associated factors and experiences of combined use”, we used the questions below.*

23. Do you use any drug to potentiate or mitigate (increase or reduce) the effect of crack?

33. What do you do to reduce or avoid the unpleasant effects of crack (for example, paranoia, craving, etc.)?
